# Perception of COVID-19 Physical Distancing Effectiveness and Contagiousness of Asymptomatic Individuals: Cross-sectional Survey of Deaf and Hard of Hearing Adults in the United States

**DOI:** 10.2196/21103

**Published:** 2021-02-25

**Authors:** Raylene Paludneviciene, Tracy Knight, Gideon Firl, Kaela Luttrell, Kota Takayama, Poorna Kushalnagar

**Affiliations:** 1 Department of Psychology Gallaudet University Washington, DC United States; 2 Department of World Languages and Cultures Sam Houston State University Huntsville, TX United States; 3 Center for Deaf Health Equity Gallaudet University Washington, DC United States; 4 Department of Social Work Gallaudet University Washington, DC United States

**Keywords:** COVID-19, coronavirus, physical distancing, asymptomatic individual, social media, deaf, hard of hearing, sign language, perception, misinformation

## Abstract

**Background:**

During the COVID-19 pandemic, there has been a rapid increase in the amount of information about the disease and SARS-CoV-2 on the internet. If the language used in video messages is not clear or understandable to deaf and hard of hearing (DHH) people with a high school degree or less, this can cause confusion and result in information gaps among DHH people during a health emergency.

**Objective:**

The aim of this study is to investigate the relationship between DHH people's perception of the effectiveness of physical distancing and contagiousness of an asymptomatic person.

**Methods:**

This is a cross-sectional survey study on DHH people's perceptions about COVID-19 (N=475). Items pertaining to COVID-19 knowledge were administered to US deaf adults from April 17, 2020, to May 1, 2020, via a bilingual American Sign Language/English online survey platform.

**Results:**

The sample consisted of 475 DHH adults aged 18-88 years old, with 74% (n=352) identifying as White and 54% (n=256) as female. About 88% (n=418) of the sample felt they knew most things or a lot about physical distancing. This figure dropped to 72% (n=342) for the question about the effectiveness of physical distancing in reducing the spread of COVID-19 and 70% (n=333) for the question about the contagiousness of an infected person without symptoms. Education and a knowledge of the effectiveness of physical distancing significantly predicted knowledge about the contagiousness of an asymptomatic individual. Race, gender, and age did not emerge as significant predictors.

**Conclusions:**

This results of this study point to the strong connection between education and coronavirus-related knowledge. Education-related disparities can be remedied by making information fully accessible and easily understood during emergencies and pandemics.

## Introduction

Health disparities have been experienced by deaf and hard of hearing (DHH) people in general, as well as during the COVID-19 pandemic. DHH people often do not have equitable access to health information, especially during emergencies [1-3], which further contributes to low perceived quality-of-life outcomes and associated health disparities [4]. For DHH people who speak American Sign Language (ASL), equitable access to health information requires an interpreter to translate between spoken English and ASL, as well as captioning, particularly for information given during live broadcasts about an emergency. To date, not all COVID-19–related video media have included sign language interpreters. However, most DHH ASL speakers, particularly DHH people with a high school degree or less, depend on information presented in ASL in videos as their go-to source for health information, with Facebook being the most often used source [5]. Previous studies have shown that DHH people with a high school degree or less have lower human papillomavirus knowledge, genetic testing awareness, and HIV screening uptake, all of which can be remedied with greater accessibility of pertinent information in ASL [6-8].

While the American public tends to rely on social media to keep up with news [9], social media can also be used as an informal source of information about others’ experiences with the health care system or as a means to gain knowledge about specific health information shared by individuals. However, when health information is reproduced and shared informally without oversight by experts, misinformation can occur, including sharing of misinformation related to the ineffectiveness of certain social behaviors in reducing the spread of infections (eg, presenting inaccurate evidence against the use of masks). People who demonstrate low electronic health (eHealth) literacy often fall victim to misinformation as they are not able to verify the accuracy of health information. They may also dismiss information about the severity of certain diseases that was shared by reliable sources on the internet [10-13]. Although the prevalence of misinformation about health on social media is harmful, it can be difficult to correct this misinformation [14]. Consequently, DHH people who have low eHealth literacy and rely on social media to learn information about COVID-19 may be at risk for believing misinformation regarding health and health risks.

During the COVID-19 pandemic, there has been a rapid increase in the amount of information about COVID-19 on the internet, resulting in an “infodemic.” This infodemic makes the identification of accurate COVID-19 information from reliable sources difficult [15-17]. DHH people who do not have the requisite background and training in emergency preparedness may select certain information to translate into ASL and share these ASL videos on social media for other DHH people to watch. If the content relayed in these videos is inaccurate or the language used is not clear or understandable to DHH people with a high school degree or less, it can cause confusion and result in information gaps among DHH people during a health emergency. These information gaps can cause harm in medically underserved DHH groups (eg, low education, low income, and low literacy) that are already at risk for disparate outcomes [18]. Accurate critical health information may also not be adequately disseminated to DHH people whose primary language is ASL. These concerns suggest a need to gather a baseline of DHH people’s awareness of and risk perceptions related to the current pandemic. Specific research aims for this study include investigating the following: (1) the relationships between DHH people’s awareness of physical distancing and perception of the effectiveness of physical distancing in reducing the spread of COVID-19, and (2) the relationship between DHH people's perceived effectiveness of physical distancing and perceived contagiousness of an asymptomatic person.

## Methods

A COVID-19 knowledge and risk perception survey in ASL was created by a team of experts in DHH health, translation, and survey development [19,20]. The web link to participate in this survey was disseminated via social media as well as through direct email invitations to those who previously took surveys through the Center for Deaf Health Equity at Gallaudet University.

### Data Collection Procedure

Following Institutional Review Board approval by Gallaudet University (RB-FY20-80), research staff recruited DHH individuals throughout the United States, both on social media and through email invitations to past survey participants. Participation in this study required access to a computer and the internet to complete an online bilingual ASL/English survey about knowledge of physical distancing and asymptomatic individuals’ contagiousness. As a result, the findings are limited to DHH people with access to computer technology and internet/Wi-Fi connectivity. Only those who self-reported using ASL as their primary language were included because this group was identified as a medically underserved group [21-23]; exclusion criteria included those aged <18 years and those who lost their hearing ability due to aging. After the participant viewed the study information in ASL and English, they were directed to a page where they could choose to provide consent or decline to participate. Following consent, the online survey presented in both ASL and English took approximately 10 minutes to complete. No names or identifying information were included in the survey and a unique identifier was used to avoid storing personal information in the survey data set.

### Participant Sources

Participants for this study were drawn from two sources: anonymous participation through a survey link shared via Facebook and a recruitment database pool that was sent an invitation email to take the online survey. The COVID-19 knowledge and risk perception survey included questions about physical distancing awareness, knowledge of physical distancing effectiveness, and knowledge of the contagiousness of an infected person without symptoms. The survey was administered to US Deaf adults from April 17, 2020, to May 1, 2020. Prior to survey administration, all items were translated into ASL with coaching from a Deaf researcher with expertise in translating survey items; the final translations were captured on film.

### Survey Items

For the purposes of this study, awareness of physical distancing and perceived effectiveness of physical distancing were assessed with the following questions: “How much do you know about physical distancing?” and “How effective do you think physical distancing is for the prevention of the spread of the coronavirus (in other words: to what extent does your physical distancing behavior contribute to other people not getting sick)?” The response options for the former question were “never knew,” “little,” “some,” “most,” and “know a lot,” while the response options for the latter question were “not at all effective,” “little effective,” “somewhat effective,” “mostly effective,” and “very effective.” Participants’ perception of the ability of an infected person without symptoms to spread COVID-19 was assessed with the following question: “Based on what you know, how contagious is someone who has been infected with the coronavirus but who shows no symptoms (no coughing; no fever)?” For this question, the response options were “not at all contagious,” “little contagious,” “somewhat contagious,” “contagious,” and “very contagious.”

### Statistical Analyses

Weighted descriptive statistics, such as cross-tabulation and percentage procedures, were used to summarize the sample. As a logistic regression model required binary response variables, responses to the perceived effectiveness of physical distancing and perceived contagiousness of an infected person without symptoms questions were recoded into binary low and high groups. Response options of “not at all effective,” “little effective,” and “somewhat effective” were recoded into the low group, while “mostly effective” and “very effective” were recoded into the high group. Response options of “not at all contagious,” “little contagious,” and “somewhat contagious” were recoded into the low group, while “contagious” and “very contagious” were recoded into the high group. A backward elimination variable selection procedure was performed on a logistic regression model, using age, gender, race, education, and baseline physical distancing awareness to predict the odds of having an accurate perception of the effectiveness of physical distancing in reducing the spread of COVID-19 (reference group). The criteria for retaining predictors was a *P* value <.05. Finally, all variables including physical distancing awareness and knowledge of physical distancing effectiveness were entered as explanatory sets to assess their relationships with knowledge of the contagiousness of an infected person without symptoms (high knowledge of the level of contagiousness was the reference). For observational, cross-sectional studies, a minimum sample size of 400 is recommended for analyses with six predictor variables in the final model [24]. SPSS (Version 25.0; IBM Corp) was used for all analyses.

## Results

The weighted sample of those that answered all questions for this study consisted of 475 adults aged 18-88 years old, with 74% (n=352) identifying as White and 54% (n=256) as female (Table 1). When asked about their preferred language on a daily basis (response options: ASL, English, both ASL and English), half of the sample (50%) preferred ASL only. About 88% (n=418) of the sample felt they knew most things about or a lot about physical distancing. This figure dropped to 72% (n=342) for the question about the effectiveness of physical distancing in reducing the spread of COVID-19 and 70% (n=333) for the question about the contagiousness of an infected person without symptoms. Bivariate correlation analysis indicated positive associations among physical distancing awareness, knowledge of physical distancing effectiveness, and knowledge of contagiousness variables (*r*=0.24-0.38; *P<*.001).

**Table 1:** Weighted sample characteristics and logistic regression of perceived contagiousness in asymptomatic individuals.

Sample characteristics		Participants, n (%)	Adjusted odds ratio (95% CI)	*P* value
**Age group (years)**				.31
	18-34	113 (23.8)	1.00	N/A^a^
	35-49	154 (32.4)	0.65 (0.29-1.43)	N/A
	50-64	126 (26.5)	0.45 (0.20-1.01)	N/A
	65-74	58 (12.3)	0.78 (0.29-2.12)	N/A
	≥75	24 (5.0)	1.17 (0.23-5.97)	N/A
**Gender identity**				.22
	Male	203 (42.8)	1.00	N/A
	Female	256 (53.8)	1.63 (0.92-2.87)	N/A
	Nonbinary	16 (3.4)	2.06 (0.24-17.94)	N/A
**Race/ethnicity**				.74
	White	352 (74.2)	1.00	N/A
	Black	21 (4.4)	0.61 (0.21-1.79)	N/A
	Latinx	67 (14.2)	0.92 (0.34-2.49)	N/A
	Asian/Other	35 (7.3)	1.26 (0.53-3.02)	N/A
**Education**				.001
	High school degree	176 (37.0)	1.00	N/A
	Some college	175 (36.8)	1.85 (0.80-4.30)	N/A
	College degree	124 (26.2)	4.67 (2.22-9.80)	N/A
Physical distancing awareness		N/A	1.66 (0.64-4.32)	.30
Physical distancing effectiveness		N/A	3.13 (1.78-5.50)	.001

^a^N/A: not applicable.

In the first model, a backward elimination variable selection procedure was used to identify the significant predictors for perceiving physical distancing as effective in reducing the spread of COVID-19. In this model, physical distancing awareness and education emerged as significant predictors of perceiving physical distancing as effective (adjusted odds ratio for physical distancing awareness=5.00, 95% CI 2.09-11.95; adjusted odds ratio for education=1.89, 95% CI 1.13-3.16). Race, gender, and age did not predict perceptions of the effectiveness of physical distancing. In the second model, both physical distancing awareness and perception of the effectiveness of physical distancing were entered as a set in the first block. All demographics were entered as a set in the second block. As shown in Table 1, with adjustments for correlates, the results showed that only perception of the effectiveness of physical distancing and education were significant in their relationships with perceived contagiousness of asymptomatic individuals. Compared to DHH respondents who had 12 years of education or lower, DHH respondents with a college degree were nearly five times more likely to report that asymptomatic individuals are contagious (adjusted odds ratio=4.67, 95% CI 2.22-9.80). Again, age, gender, and race did not predict knowledge of contagiousness of an infected person without symptoms.

## Discussion

### Principal Findings

A majority of this US DHH study sample had heard of physical distancing, but not all believed that physical distancing was effective in reducing the spread of COVID-19 or that an asymptomatic individual was contagious. Above and beyond sociodemographic variables and physical distancing awareness, DHH people who had a college degree were almost twice as likely to believe that physical distancing was effective at reducing the spread of COVID-19 compared to DHH people who did not have a college degree. Although both education and physical distancing awareness strongly predicted perceptions of the effectiveness of physical distancing, this study did not find evidence for the contribution of age, gender, and race to the development of appropriate perception of physical distancing effectiveness. This finding is consistent with research studies that reported a connection between more years of education and a greater level of health-related awareness or screening uptake [6-8]. Our study also showed that DHH people who have a college degree, as well as knowledge of the effectiveness of physical distancing, were much more likely to believe that an infected person without symptoms is contagious compared to DHH people who did not have a college degree and demonstrated low knowledge of the effectiveness of physical distancing.

The lower knowledge of physical distancing and COVID-19 among DHH people who do not have a college degree may be in part explained by inadequate, inaccessible, or misleading information presented in ASL on social media. According to the 2020 World Health Organization situation report on the novel coronavirus, the COVID-19 outbreak has been accompanied by a global infodemic on social media. As a result, some social media sources may be of low quality, which can potentially lead to the sharing of unreliable information by people in the community. At the same time, social media has been cited as an essential tool for health information seeking among DHH people who do not have a college degree [5] and it can reliably be used to promote rational social health behavior during emergencies and pandemics. Thus, for all social media postings, ASL videos about health during a pandemic must actively cite and include trusted sources for further information. Given the strong connection between education and eHealth literacy [25], as well as the importance of the readability of online health content to accommodate people with a low level of education [26], our community has a responsibility to ensure that social media health videos are fully accessible in ASL and understood by all, including those who have only a high school degree. Clinicians have a responsibility to incorporate patient-centered care and communication in their practice to ensure that patients who are DHH understand what they need to do to take care of their health. Next, we provide recommendations on the development of videos to help increase ASL comprehension for DHH people with a high school education or less.

DHH people with limited access to written English due to language delay or deprivation and DHH immigrants whose first language is not English often have limited ASL fluency [27]. To best accommodate the language needs of these underserved subgroups, ASL videos should be screened for readability for DHH with a high school education or less. This may include avoiding relying on fingerspelling to describe concepts, and instead using visual descriptions and illustrations to make the information easier to understand. Specific concepts might need more elaboration, such as how germs are spread and how masks help prevent the spread of germs. Increasing the use of visual descriptions and elaborating on complex concepts would be key components in providing clear and well-conceived emergency messages. Videos in ASL that include visual descriptions and elaboration of concepts are likely to support comprehension, be effectively transmitted, and ultimately help ensure public health safety by reducing the spread of COVID-19.

According to the National Association of the Deaf Position Statement on Accessible Emergency Management for Deaf and Hard of Hearing People [28], the best practices to disseminate awareness training to DHH citizens during the pandemic are the following: (1) ensuring that the materials are accessible in a language that they understand, (2) including medical and public health experts who use ASL in interactive panels, (3) providing training to qualified sign language interpreters, and (4) ensuring that all news and notification systems are accessible by any form of telecommunication technology.

### Conclusion

The results of this study point to the strong connection between education and COVID-19–related knowledge. Therefore, the information that DHH organizations and public health agencies disseminate during emergencies and pandemics must be clear, contain adequate and reliable information, and be timely in concordance with other information being disseminated. For online streaming of time-sensitive news, such as state and local government updates, live captioning in addition to sign language interpreting would provide DHH people with full access to information. ASL videos that are created for social media or the internet must include features that support ease of understanding, which fosters improved knowledge and more accurate perceptions. Information in ASL disseminated through other sources (such as television and the news) must also be clear and accessible to the whole DHH community.

## Abbreviations

ASL: American Sign Language
DHH: deaf and hard of hearing
eHealth: electronic health
